# Dermatologic, Orthopedic, and Cardiovascular Manifestations and Management in a Geriatric Patient With Dermatosparaxis-Type Ehlers-Danlos Syndrome: A Case Report

**DOI:** 10.7759/cureus.73211

**Published:** 2024-11-07

**Authors:** Assem Al Sayed, Chrystal Sumrall

**Affiliations:** 1 Family Medicine, William Carey University College of Osteopathic Medicine, Hattiesburg, USA; 2 Family Medicine, Hattiesburg Clinic, Laurel, USA

**Keywords:** aortic aneurysm repair, bruisability, dermatosparaxis, ehlers-danlos syndrome (eds), generalized joint hypermobility, mitral valve prolapse, skin fragility

## Abstract

Ehlers-Danlos syndrome (EDS) is a diverse group of hereditary connective tissue disorders resulting from mutations in genes involved in the synthesis and metabolism of collagens. Collagen, a structural protein in the connective tissues, plays an important role in maintaining the integrity and strength of various tissues, including the skin, ligaments, tendons, cartilage, and blood vessels. As such, EDS is characterized by joint hypermobility, skin elasticity, and tissue fragility. This paper discusses the case of an elderly patient with dermatosparaxis-type EDS (dEDS), a rare autosomal recessive subtype caused by mutations in the ADAMTS2 gene, leading to significant skin fragility, among other characteristic manifestations. This case highlights the complexities involved in the management of the diverse dermatological, orthopedic, and cardiovascular manifestations of dEDS and underscores the importance of individualized care plans that address the complexities of dEDS and improve the quality of life of dEDS patients.

## Introduction

Ehlers-Danlos syndrome (EDS) is a heterogeneous group of inheritable connective tissue disorders that mainly involves the cardiovascular and musculoskeletal systems [1]. EDS primarily results from mutations in genes involved in the synthesis and metabolism of collagen, a fundamental structural protein in the connective tissues that plays a critical role in maintaining the integrity and strength of the skin, ligaments, tendons, cartilage, and blood vessels [1,2]. EDS manifests clinically as varying degrees of joint hypermobility, skin elasticity, tissue fragility, and abnormal wound healing [1]. Originally classified into six types by the 1998 Villefranche classification system, EDS classification has evolved as our analysis tools and understanding of the condition’s genetic and biochemical causes advanced [1]. The extended 2017 classification identifies 13 distinct clinical subtypes of EDS, with 20 genes identified to be associated with the condition [1]. Each subtype is defined by specific clinical features, genetic mutations, and inheritance patterns [1].

Dermatosparaxis-type EDS (dEDS), also known as EDS VIIC, is a rare, autosomal recessive type of EDS, characteristically manifesting clinically with severe skin fragility [2-4]. dEDS arises from mutations in the ADAMTS2 gene as a result of homozygosity or compound heterozygosity of mutant alleles [4]. The ADAMTS2 gene encodes for procollagen I N-proteinase, a critical enzyme in the correct processing of type I procollagen into type I collagen, a major structural protein in the extracellular matrix [1,4,5]. This defective collagen forms ribbon-like fibrils that are unable to support the structural integrity or provide the normal tensile strength of the skin and other connective tissues [5]. Consequently, individuals with dEDS experience profound skin fragility and sagging [6].

The clinical diagnosis of dEDS is made based on meeting certain major and minor criteria from the extended 2017 classification and confirmed with genetic testing [1]. The management of EDS is mainly supportive, focusing primarily on symptomatic management. A multidisciplinary approach is crucial to providing comprehensive care and improving the quality of life for those affected by this syndrome [7]. In this study, we present the case of an elderly male patient with dEDS and address the management of his dermatologic, orthopedic, and cardiovascular manifestations.

## Case presentation

A 68-year-old Caucasian male presented to the clinic to establish care. His contributory past medical history was significant for primary hypertension, primary osteoarthritis of the right knee, and carpal tunnel syndrome of the right wrist. His history revealed an allergy to sulfa drugs, for which he developed a generalized rash. His contributory past surgical history was significant for right carpal tunnel decompression surgery and total knee arthroplasty of the right knee. His history was also significant for cigarette smoking; however, he has not smoked for the past 11 years. His family history was significant for cancer and chronic kidney disease in his father and cancer in his mother.

On examination, the patient appears well-developed and well-nourished without acute distress. His vital signs were blood pressure 142/89 mmHg, heart rate 87 beats per minute, temperature 97 °F, respiratory rate 20 breaths per minute, height 5′5″, weight 181 lbs, body mass index (BMI) 30.12, and oxygen saturation 98% on room air. The cardiovascular examination showed regular heart rate and rhythm, heart sounds dual, and without any murmurs, clicks, snaps, gallops, or friction rubs. Similarly, pulmonary, abdominal, neurologic, and psychiatric examinations revealed no significant findings or deficits. The musculoskeletal examination showed normal muscle tone; however, the physical examination revealed generalized joint hypermobility. Furthermore, examination of the right knee demonstrated a full range of motion without any evidence of subluxation or adhesive capsulitis. The dermatologic examination was significant for redundant, lax skin with excessive skin folding, especially around the forehead, eyes, and forearms. On palpation, the patient’s skin was hyperextensible with a soft, doughy texture and demonstrated extreme fragility, as evident by the generalized petechiae and subcutaneous hematomas. The patient also presented with puffy eyelids, prominent epicanthal folds, and down-slanting palpebral fissures.

Given the patient’s symptomology and presentation, the clinical diagnosis of dEDS was made. A complete blood count, basic metabolic panel, and lipid panel were ordered, the results of which did not display any significant findings. Given his current age, past history of cigarette smoking, and suspected diagnosis, a duplex ultrasound was ordered to screen for an abdominal aortic aneurysm, which was negative for dilation or aneurysm of the aorta and right and left common iliac arteries. Additionally, a point-of-care 12-lead electrocardiogram was ordered to check for left atrial enlargement or left ventricular hypertrophy which would raise suspicion for mitral valve prolapse; however, it too revealed no abnormalities.

Following the patient’s high blood pressure reading at the visit, ambulatory blood pressure monitoring was ordered, which revealed diastolic blood pressure readings that were consistently in the 90s and 100s. As such, the patient was placed on triamterene-hydrochlorothiazide (37.5-25 mg daily), which controlled his hypertension. His blood pressure readings at six- and 12-month follow-up appointments were 112/64 and 117/60 mmHg, respectively.

## Discussion

The clinical diagnosis of dEDS is made based on the criteria from the extended 2017 classification and confirmed with genetic testing [1]. Table 1 lists the major and minor clinical criteria for patients with dEDS [1,3,4,6]. To meet the minimum diagnostic criteria for dEDS, a patient must present with extreme skin fragility (major clinical criterion 1) and characteristic craniofacial features (major clinical criterion 2) in addition to one other major clinical criterion or three minor clinical criteria [1,3,4,6].

**Table 1:** Major and minor clinical criteria for the diagnosis of Ehlers-Danlos syndrome, dermatospraxis type. ^a^Characteristic craniofacial features include prominent and protuberant eyes with puffy, edematous eyelids, epicanthal folds, down-slanting palpebral fissures, blue sclerae, large fontanels, delayed fontanel closure, and hypoplastic chin. ^b^Characteristic craniofacial features are evident at birth, early infancy, or later in childhood.

Major clinical criteria	Minor clinical criteria
1) Extreme skin fragility with congenital or postnatal tears	1) Soft and doughy skin texture
2) Characteristic craniofacial features^a,b ^	2) Skin hyperextensibility
3) Redundant, almost lax skin, with excessive skin folds at the wrists and ankles	3) Atrophic scars
4) Increased palmar wrinkling	4) Generalized joint hypermobility
5) Severe bruisability with risk of subcutaneous hematoma	5) Complications of visceral fragility, such as bladder or diaphragmatic rupture
6) Umbilical hernia	6) Delayed motor development
7) Postnatal growth retardation	7) Osteopenia
8) Short limbs	8) Hirsutism
9) Perinatal complications due to connective tissue fragility	9) Tooth abnormalities
	10) Refractive errors
	11) Strabismus

This patient’s presentation with four of the nine major clinical criteria and three of the 11 minor clinical criteria qualifies him for a clinical diagnosis of dEDS, as per the diagnostic criteria proposed by Malfait et al. [6]. Namely, this patient presented with extreme skin fragility (major clinical criteria 1); prominent eyes with puffy, edematous eyelids, epicanthal folds, and down-slanting palpebral fissures (major clinical criteria 2); redundant, lax skin (major clinical criteria 3); severe bruising manifesting as generalized petechiae and purpura (major clinical criteria 5); soft and doughy skin texture (minor clinical criteria 1); skin hyperextensibility (minor clinical criteria 2); and generalized joint hypermobility (minor clinical criteria 4).

Dermatologic manifestations and management

In dEDS, the skin exhibits a greater degree of impairment compared to other collagen-rich tissues [4], as evident in the patient’s presentation. In addition, the patient’s presentation with the characteristic puffy eyelids and excessive periorbital skin [4] further supports the diagnosis of dEDS. Unlike cutis laxa, which also presents with facial skin redundancy, dEDS presents with skin fragility and dermal bruising [3], features that are present in this patient. Our patient, however, lacked any atrophic scars, extensive scarring, or poor wound healing, all of which are typical features of dEDS [3,4]. As skin fragility is directly related to the concentration of collagen precursors, skin fragility decreases as a function of age due to the increasing density of collagen fibers in the skin during development and slower collagen turnover [4].

The management of the dermatologic manifestations of EDS involves the utilization of preventative measures that limit the skin injury, including avoidance of wound tension, use of low-adherence dressings, avoidance of high-impact sports, and use of protective equipments, such as shin guards [8]. Additionally, in patients who present with bruising, compression bandages and pharmacologic treatment ought to be considered in the management plan [8].

Orthopedic manifestations and management

The increased length and elasticity of the connective tissues seen in EDS are responsible for the increased range of motion and distractibility of joints, which in turn elevate the risk of orthopedic complications such as subluxation, dislocation, and tendon rupture [9]. Joint laxity, in particular, seems to become a major clinical feature with increasing age [4]. Additionally, as seen with the patient’s presentation, knee instability is a common orthopedic manifestation associated with EDS [10]. The hypermobility of the knee joint increases the risk of developing meniscal and ligamentous tears [10]. Furthermore, the joint instability and asymmetric loading of joint surfaces during subluxation accelerate the wear of the joint’s surfaces, predisposing to osteoarthritis [4,10].

The management of orthopedic complications in patients with EDS requires a multidisciplinary team approach composed of orthopedic surgeons, rheumatologists, pain consultants, and occupational and physiotherapists [10]. Nonsurgical management options in these patients ought to be exhausted prior to surgical interventions due to the potential for postsurgical complications secondary to abnormal connective tissue healing and muscular deconditioning [7,10]. Nonsurgical management should include low-impact, low-resistance exercise programs, such as aquatic therapy, that emphasize joint stability, isometric and eccentric muscle strengthening, proprioception, and improved posture [7,9,10]. Additionally, physiotherapy directed toward strengthening the core muscles and stabilizing the spine, shoulder, and knees can significantly improve outcomes [10]. The use of supportive devices such as braces may be helpful in reducing joint instability as well [9].

Cardiovascular manifestations and management

Cardiovascular manifestations in patients with EDS can range from no observable lesions to arterial aneurysms, varicose veins, mitral valve prolapse (MVP), mitral or tricuspid regurgitation, and cardiac conduction disturbances [9,11]. Thus, echocardiograms are warranted in patients with EDS [9]. Additionally, if a patient with EDS presents with MVP and regurgitation, prophylactic antibiotics for bacterial endocarditis are indicated [11].

Collagen and elastin are crucial components of the large arteries as they determine their passive mechanical properties [12]. Collagen, which is stiffer, is load-bearing at high pressures, whereas elastin, which is distensible, is load-bearing at low pressures [12]. Thus, changes in the quality or ratio of collagens alter the wall mechanics of these large arteries [12]. Furthermore, in their study, Miller et al. concluded that increased arterial elasticity may be related to impaired blood pressure control in EDS [13]. Thus, regular imaging may be warranted in these patients given the increased risk of aortic dilation [9].

Finally, bruising in these patients is due to vessel wall fragility as a result of the poor connective tissue support in the vessel wall, rather than abnormal platelet or coagulation function, and laboratory coagulation studies in these patients often return normal [9]. Of note, intramuscular injections are discouraged in patients with EDS due to vessel wall fragility and potential ecchymosis [9].

## Conclusions

This case illustrates the presentation and management of the complexities of dEDS in a geriatric patient, highlighting the significant dermatological, orthopedic, and cardiovascular manifestations involved. A multidisciplinary approach is essential to address the diverse needs of individuals with dEDS to ensure optimal symptomatic management and improved quality of life. Continued research and clinical awareness of this rare condition are necessary to enhance our understanding of this condition and its long-term complications.
